# Neurological complications during veno-venous extracorporeal membrane oxygenation: Does the configuration matter? A retrospective analysis of the ELSO database

**DOI:** 10.1186/s13054-021-03533-5

**Published:** 2021-03-17

**Authors:** Roberto Lorusso, Mirko Belliato, Michael Mazzeffi, Michele Di Mauro, Fabio Silvio Taccone, Orlando Parise, Ayat Albanawi, Veena Nandwani, Paul McCarthy, Zachary Kon, Jay Menaker, Daniel M. Johnson, Sandro Gelsomino, Daniel Herr

**Affiliations:** 1 Cardio-Thoracic Surgery Department, Heart and Vascular Centre, Maastricht University Medical Centre, Maastricht, The Netherlands; 2 Departments of Anesthesiology, University of Maryland School of Medicine, Program in Trauma, R Adams Cowley Shock Trauma Center, Baltimore, USA; 3 Departments of Surgery, University of Maryland School of Medicine, Program in Trauma, R Adams Cowley Shock Trauma Center, Baltimore, USA; 4 Cardiac Surgery Unit, University of Chieti, Chieti, Italy; 5 UOC Anestesia e Rianimazione 1, Foundation IRCCS Policlinico San Matteo, Pavia, Italy; 6 Department of Cardiothoracic Surgery, NYU Langone Health, New York, NY USA; 7 Department of Intensive Care, Erasme Hospital, Université Libre de Bruxelles (ULB), Route de Lennik, 808, 1070 Brussels, Belgium

**Keywords:** Extracorporeal membrane oxygenation, V-V ECMO, Neurological complications, Respiratory failure, Cannulation, Seizure, Propensity score

## Abstract

**Background:**

Single- (SL) and double-lumen (DL) catheters are used in clinical practice for veno-venous extracorporeal membrane oxygenation (V-V ECMO) therapy. However, information is lacking regarding the effects of the cannulation on neurological complications.

**Methods:**

A retrospective observational study based on data from the Extracorporeal Life Support Organization (ELSO) registry. All adult patients included in the ELSO registry from 2011 to 2018 submitted to a single run of V-V ECMO were analyzed. Propensity score (PS) inverse probability of treatment weighting estimation for multiple treatments was used. The average treatment effect (ATE) was chosen as the causal effect estimate of outcome. The aim of the study was to evaluate differences in the occurrence and the type of neurological complications in adult patients undergoing V-V ECMO when treated with SL or DL cannulas.

**Results:**

From a population of 6834 patients, the weighted propensity score matching included 6245 patients (i.e., 91% of the total cohort; 4175 with SL and 20,270 with DL cannulation). The proportion of patients with at least one neurological complication was similar in the SL (306, 7.2%) and DL (189, 7.7%; odds ratio 1.10 [95% confidence intervals 0.91–1.32]; *p* = 0.33). After weighted propensity score, the ATE for the occurrence of least one neurological complication was 0.005 (95% CI − 0.009 to 0.018; *p* = 0.50). Also, the occurrence of specific neurological complications, including intracerebral hemorrhage, acute ischemic stroke, seizures or brain death, was similar between groups. Overall mortality was similar between patients with neurological complications in the two groups.

**Conclusions:**

In this large registry, the occurrence of neurological complications was not related to the type of cannulation in patients undergoing V-V ECMO.

**Supplementary Information:**

The online version contains supplementary material available at 10.1186/s13054-021-03533-5.

## Introduction

Veno-venous (V-V) extracorporeal membrane oxygenation (ECMO) can be lifesaving in severe respiratory failure [1]. According to Extracorporeal Life Support Organization (ELSO), the reported survival rate is 66%, when considering more than 15,000 treated adult patients [2]. In particular, young patients with severe respiratory failure, who are typically treated during the influenza epidemics, may demonstrate even major benefits when V-V ECMO is implemented [3, 4].

However, the use of V-V ECMO is not without risks and neurologic complications have been reported, although their incidence is lower than in patients undergoing veno-arterial (V-A) ECMO [5–7]. According to a recent retrospective cohort study from the Extracorporeal Life Support Organization (ELSO) Registry including 4,988 patients treated with V-V ECMO, 356 (7%) of them suffered from neurological complications, in particular intra-cranial hemorrhage (42.5%), stroke (19.9%), seizures (14.1%) and brain death (23.5%) [8]. The current mechanistic understanding of neurologic injury during V-V ECMO is limited. Several putative risk factors have been proposed, including acute renal failure and a rapid PaCO_2_ decrease at the time of ECMO cannulation [5]. Cerebral micro-emboli have also been detected in patients undergoing both V-V and V-A ECMO and may play a role in the cerebrovascular injury [9]. Another potential contributing factor to neurologic injury during V-V ECMO is cerebral venous congestion, which may be caused by large cannulas in the internal jugular veins or venous thrombosis [10, 11, 13]. This phenomenon has been also confirmed in an animal ECMO models [12].

V-V ECMO is currently performed using either single- (SL) or dual-lumen (DL) cannulas. DL V-V ECMO cannulas have several potential advantages, including single vessel cannulation, facilitation of ambulation and less recirculation [14, 15]. However, DL V-V ECMO cannulas are also characterized by larger sizes (i.e. 27–31 French in most of cases) than SL, which might predispose patients to cerebral venous congestion [15]. A previous single-center observational study conducted in patients undergoing V-V ECMO with DL cannulation reported a rate of intracranial hemorrhage of 7%; only 20% of these patients survived to hospital discharge [16]. In an additional study focusing on a pediatric population on V-V ECMO, no differences were observed in the total complications and survival rate between SL and DL cannulations [17]. Interestingly, there was a nonsignificant trend towards a lower rate of neurological complications in the SL group.

Taking all these data into account, we hypothesize that the use of DL in V-V ECMO patients may be associated with a higher rate of neurologic injury. For these reasons, we evaluated the occurrence and the type of neurologic complications in a large cohort of adult patients on V-V ECMO, according to the SL or DL cannulation.

## Methods

### Study design, setting and participants

This is a retrospective study including adult patients undergoing V-V ECMO from 2011 to 2017. All data were extracted from the ELSO database (until 2018). Patients supported with V-A ECMO, those supported with VV-ECMO for cardiac indications, and those supported with multiple ECMO runs were excluded. Patients less than 18 years of age were also excluded. The ELSO registry collects data on all ECMO cases from approximately 800 centers around the world since 1989. Data are collected using a standardized data collection form. Data user agreement between ELSO and member centers allows the use of deidentified datasets for research without need for further regulatory approval.

### Collected variables

For all patients, we collected the following variables from the ELSO database: age, gender, weight, cannulation type (SL vs. DL), total ECMO hours, pre-ECMO arrest, fraction of inspired oxygen (FiO_2_) before ECMO, peak inspiratory pressure before ECMO, positive end expiratory pressure before ECMO, pH before ECMO, PaCO_2_ before ECMO, PaO_2_ before ECMO, serum bicarbonate level before ECMO, intubation hours before ECMO, and pump flow at 4 h and 24 h after ECMO initiation.

### Study end point

The primary end point of the study was to evaluate the occurrence of neurological complications in the SL and DL group. ELSO registry categorizes “neurologic injury” as intracranial hemorrhage (ICH), acute ischemic stroke (AIS), seizures (either clinical and/or on electroencephalography, EEG) and brain death (BD) that occur during the ECMO run. All the cases are also confirmed, at least for ICH and AIS, by a neurologist and serial CT scans. Additional data on severity, site, timing and functional long-term neurologic recovery were not available. Secondary outcomes included the type of neurological complications (i.e. hemorrhagic stroke, ischemic stroke and seizure) in the two groups.

### Statistical analysis

Propensity score (PS) inverse probability of treatment weighting estimation for multiple treatments was used. The balance was tested either graphically or utilizing balance tables. Graphical estimation used standardized effect plots and quantile–quantile plots, which provide an immediate visual evaluation of balance quality. In each model, absolute standardized mean differences (ASMD) were calculated using a cutoff of less than 0.10 for bias statistics. The average treatment effect (ATE) was chosen as the causal effect estimate of each outcome. It was defined as the ratio of the incidence of the outcome in the entire population undergoing one treatment over the impact of the outcome of the whole population under another treatment [18].

Inverse Probability of Treatment Weighting (IPTW) was used to correct for imbalances between groups on pre-treatment covariates so that the distribution of the pre-treatment characteristics would be similar across all the groups. A machine learning technique, generalized boosted model (GBM), was used to estimate the PS weights. GBM estimation captures complex relationships between treatment assignment and pre-treatment variables without over-fitting data, and GBM can be fine-tuned to find the best balance among groups. IPTW is one technique for reducing the bias due to observed variables. It relies on two key conditions for obtaining unbiased estimates 1: (1) No unknown or unmeasured confounders assumption or exchangeability and (2) Sufficient overlap or positivity: 0 < Pr (Ti = *t*|*X*) < 1, for all *X* and *t*, where Ti is the random treatment assignment variable, Pr is probability, *X* is the vector of observed treatment covariates and *t* is the treatment. The first assumption states that the set of observed variables is rich enough to include all variables influencing both treatments and outcomes. The second condition states that each patient has a non-zero probability of receiving each treatment. Both assumptions were met in our models.

For our outcomes, many simple regression trees were generated starting from a single regression tree and adding another tree at each new iteration to create an overall piecewise constant function. This iterative fitting algorithm was chosen so as to provide the best fit to the residuals of the model from the previous iteration and because it offers the greatest increase to the log likelihood for the data. Indeed, each iteration increases the likelihood making the model sufficiently flexible to perfectly fit data. To avoid data overfitting, GBM selects an intermediate iteration (or number of trees) for the final model so as to “minimize an external criterion such as out-of-sample prediction error or—in the case of propensity score estimation—imbalance on the pre-treatment covariates across the treatment and control groups. Therefore, the key is to use GBM iteratively with the optimal iteration (number of trees) for estimating the PS and minimizing a “stopping rule” criterion based on the difference between the weighted distributions of the pre-treatment variables in the two treatment conditions. In practice, different stopping rules have been used to select the optimal iteration of GBM for use in estimating propensity score weights: maximum or minimum absolute standardized bias (SB, also referred to as the absolute standardized mean difference) or the Kolmogorov–Smirnov (KS) statistic, each of which compares the means or the distributions of the covariates between treatment groups. Since the balance was nearly invariant with the stopping rule, we used the max Kolmogorov–Smirnov (KS) statistic.

For 2 groups the KS is:

$$\left[\kern-0.15em\left[ {KS} \right]\kern-0.15em\right]\_k = \left[\kern-0.15em\left[ {\sup } \right]\kern-0.15em\right]\_x \, \left| {EDF1k\left( x \right){-}EDF0k\left( x \right)} \right|$$

where EDF is the empirical distribution function for the treatment and control samples and *k* is the covariate. Causal effects can be estimated through two different summaries: average treatment effect (ATE) and average treatment effect between treatments (ATT). The ATE of treatment “ti” versus treatment “tj” is the comparison of mean outcome had the entire population been observed under treatment “ti” versus had the entire population been observed under treatment “ti”. The ATT of “ti” versus “tj” is the comparison of the mean “ti” patient outcome with the mean outcome they would have had if they had instead been treated by “tj” treatment. The casual effect was estimated by ATE which takes into account summary statistics of the effects across populations of interest.

R software v. 3.6.1 (R Foundation for Statistical Computing, Vienna, Austria) and the TWANG and SURVEY packages were used for analysis.

## Results

### Study population

A total of 6,834 patients met the inclusion criteria; of those, 4367 (63.9%) had SL cannulation and 2467 (36.1%) had DL cannulation. Table 1 shows characteristics of the study population by cannulation type. Interestingly, patients who underwent SL cannulation were older, less likely female, had fewer total ECMO hours and lower body weight. Despite similar ventilatory parameters prior to ECMO, peak inspiratory pressures were slightly lower in the SL than in the DL group. Also, mean PaCO_2_, PaO_2_, and bicarbonate levels were significantly lower and pump flows higher in SL group than others.

**Table 1:** Main patients characteristics, before weighing using a propensity score

Variable	DL	SL	*p* value
	*N* = 2467	*N* = 4367	
Age (years)	46 (32–58)	48 (35–60)	< 0.0001
Male gender [*n* (%)]	1386 (56.5)	2612 (62.0)	< 0.0001
Weight (kg)	83 (70–102)	80 (68–100)	0.0001
Total ECMO hours	191 (97–362)	189 (96–336)	0.058
Pre-ECMO arrest [*n* (%)]	165 (6.7)	294 (6.7)	0.984
FiO_2_ prior to ECMO (%)	100 (100–100)	100 (100–100)	0.608
PIP prior to ECMO (cmH_2_O)	35 (30–40)	34 (30–38)	< 0.0001
PEEP prior to ECMO (cmH_2_O)	13 (10, 16)	14 (10, 16)	0.367
pH prior to ECMO	7.24 (7.14–7.33)	7.24 (7.15–7.34)	0.152
PaCO_2_ prior to ECMO (mmHg)	57 (43–75)	52 (37–69)	< 0.0001
PaO_2_ prior to ECMO (mmHg)	59 (45–75)	56 (41, 72)	< 0.0001
HCO_3_ prior to ECMO (mEq/L)	25 (21–31)	24 (20–29)	< 0.0001
Intubation hours prior to ECMO	45 (13–131)	41 (15–120)	0.166
Pump flow at 4 h (L/min)	4.0 (3.4–4.5)	4.0 (3.4–4.8)	< 0.01
Pump flow at 24 h (L/min)	4.0 (3.4–4.6)	4.1 (3.4–4.8)	< 0.01
Alive at hospital discharge [*n* (%)]	1580 (64.0)	2595 (60.8)	0.002

Data are presented as count (%) or median (IQRs)

*DL *double lumen cannula, *SL *single-lumen cannula, *ECMO* extracorporeal membrane oxygenation, *PEEP* positive end expiratory pressure, *PIP* peak inspiratory pressure

After propensity score, a total of 6245 patients (i.e. 91% of the total cohort; 4175 with SL and 20,270 with DL cannulation) were included for the outcome analysis. The maximum pairwise ASMD was 0.10 for all selected variables (Additional file 1: S1–3).

### Types of neurologic injury and outcome

**Fig. 1:**
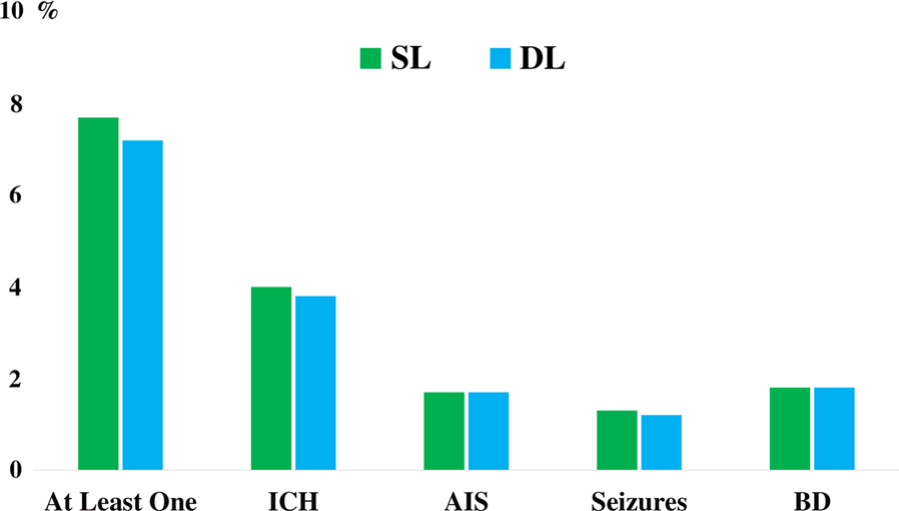
Occurrences of various neurological injuries in the study population, according to the cannulation strategy. *DL* dual lumen, *SL *single lumen

Considering the entire cohort, 306 (7.2%) patients in the SL group had at least one neurological injury: ICH in 161 patients (3.8%), AIS in 73 patients (1.7%), seizures in 52 patients (1.2%; 44/52 clinically determined) and BD in 44 patients (1.8%) (Table 2–Fig. 1). One hundred-eighty-nine (7.7%; OR 1.10 [0.91–1.32]; *p* = 0.33 vs. SL group) patients in the DL group had at least one neurological injury: ICH in 99 patients (4.0%; OR 1.09 [0.84–1.41]; *p* = 0.51 vs. SL group), AIS in 42 patients (1.7%; OR 1.01 [0.69–1.49]; *p* = 0.92 vs. SL group), seizures in 33 patients (1.3%; 27/33 clinically determined—OR 1.10 [0.72–1.67]; *p* = 0.66 vs. SL group) and BD in 44 patients (1.8%; OR 1.02 [0.71–1.48]; *p* = 0.92 vs. SL group) (Table 2; Fig. 1).

**Table 2:** Neurologic complications according to the cannulation strategy, before weighing using a propensity score

Variable	DL	SL	OR	Lower 95% CI	Upper 95% CI	*p* value
	*N* = 2467	*N* = 4367				
Patients with CNS Complications	189 (7.7%)	306 (7.2%)	1.10	0.91	1.32	0.33
ICH	99 (4.0%)	161 (3.8%)	1.09	0.84	1.41	0.51
AIS	42 (1.7%)	73 (1.7%)	1.01	0.69	1.49	0.92
Seizures	33 (1.3%)	52 (1.2%)	1.10	0.72	1.67	0.66
Brain death	44 (1.8%)	76 (1.8%)	1.02	0.71	1.48	0.92

*DL* double lumen cannula, *SL* single-lumen cannula, *OR* odds ratio, *CI* confidence intervals, *ICH* intracranial hemorrhage, *AIS* acute ischemic stroke, *CNS* central nervous system

When comparing DL to SL configuration, the ATE for the occurrence of least one neurological complication was 0.005 (95% CI − 0.009 to 0.018; *p* = 0.50), for ICH was 0.003 (95% CI − 0.007 to 0.013; *p* = 0.50), for AIS was 0.001 (95% CI − 0.007 to 0.007; *p* = 0.95), for seizures was 0.001 (95% CI − 0.005 to 0.006; *p* = 0.81) and for BD was − 0.002 (− 0.008 to 0.005; *p* = 0.63).

The overall survival rate was lower in the DL than the SL group (64.0% vs. 60.8%, *p* = 0.002—Table 3—ATE: 0.033 [0.008–0.059]; *p* = 0.01). However, survival was similar among DL and SL among all the subgroups of neurological injuries (Table 3). When comparing DL to SL configuration, the ATE for survival in patients with at least one neurological complication was − 0.046 (− 0.127 to 0.035; *p* = 0.27).

**Table 3:** Patient outcomes, according to the cannulation strategy, before weighing using a propensity score

Variable	DL	SL	OR	Lower 95% CI	Upper 95% CI	*p* value
	*N* = 2467	*N* = 4367				
Discharged alive, all	1580 (64.0)	2595 (60.8)	1.13	0.96	1.34	0.002
Discharged alive, ICH	21/99 (21.0%)	35/161 (21.7%)	0.96	0.52	1.78	> 0.99
Discharged alive, AIS	7/42 (16.6%)	24/73 (32.8%)	0.41	0.16	1.03	0.08
Discharged alive, seizures	13/33 (39.4%)	26/52 (50.0%)	0.65	0.28	1.56	0.38
Discharged alive, CNS complications	38/189 (20.1%)	79/306 (25.8%)	0.72	0.46	1.11	0.16

*DL* double lumen cannula, *SL* single-lumen cannula, *OR* odds ratio, *CI* confidence intervals, *CNS* central nervous system

## Discussion

In this study based on a large international registry, we observed that DL V-V ECMO cannulation was associated with a similar occurrence of neurological complications than SL cannulation. Also, mortality was higher in the SL group, but similar across different types of neurological injury, regardless of the type of cannulation.

DL cannulation has several potential advantages over traditional cannulation, including easier ambulation and reduced recirculation. However, it remains unclear whether this approach would increase the risk of specific complications in such patients. We analyzed a large registry including ECMO centers which report routinely their data; also, using a matching method, we compared similar populations of patients undergoing V-V ECMO and receiving two different type of cannulation. In this study, DL cannulation was associated with a similar incidence of neurological complications than DL. Few data are available on the occurrence of seizures in adult patients undergoing V-V ECMO. In a recent study including 139 patients undergoing both veno-arterial (V-A) and V-V ECMO concomitantly with EEG monitoring, Peluso et al. reported an 8% occurrence of seizures or status epilepticus [19], which was independent from ECMO configuration. Nevertheless, no DL cannulation was used in this cohort. In a large registry analysis (*N* = 4988), Lorusso et al. reported 60 patients with seizures (1.2%), mostly being clinically diagnosed [7]. The use of EEG monitoring has already shown to increase the detection of seizures, which are mainly non-convulsive in critically ill patients [20]; unfortunately, in many ECMO centers continuous EEG monitoring is not routinely implemented or not available, and the real occurrence of seizures might have been largely underestimated. Also, as some seizures were “clinically determined”, it remains unknown whether they were convulsions or other forms of abnormal movements, which might occur in critically ill patients. Moreover, few studies have tried to assess the causes of epileptic complications in ECMO patients. If in critically ill patients admitted for medical causes, sepsis, drug toxicity, metabolic disturbances or discontinuation of antiepileptic drugs have been associated with a higher probability of seizures [21–23], our study was unable to assess those factors and suggested no additional role for the selection of cannulation on the occurrence of such complication.

In this study, the occurrence of other type of neurological injuries, such as intracranial hemorrhage, acute ischemic infarction and brain death, was also similar between DL than SL cannulation. A previous analysis of a French cohort consisting of 135 consecutive patients undergoing V-V ECMO showed that 14.1% had a neurologic injury [5]. The majority of these events were ICH, with only a small number of ischemic strokes or diffuse microbleeds. In the present study, with more than 2000 patients included, the total number of neurological events was less than half when compared to that previously reported [5, 7]. Of course, differences in age, indications for ECMO, use of anticoagulation, the presence of previous neurological diseases and different policies to obtain cerebral CT scan might also explain these findings. The pathophysiology of neurologic injury during V-V ECMO is complex with many processes potentially playing a role. These include frequent changes in PaO_2_ and PCO_2_, that can affect cerebral blood flow, formation of cerebral micro-emboli and venous congestion from cannulation of the internal jugular veins. Furthermore abrupt changes in local and systemic blood pressure, ischemia/reperfusion, anticoagulation and venous hypertension caused by distal internal vein ligation have also been reported to play a contributory role [24, 25]. To date, there are few detailed investigations of cerebral blood flow and cerebral venous return in ECMO patients. In one study of pediatric ECMO patients, cerebral blood flow velocities were below normal ranges [26]; the authors of this study concluded that reduced blood flow velocities could be due to the decreased cerebral metabolic demands associated with sedation, cerebral venous congestion, or reduced cardiac function during ECMO. Future studies using more precise assessment of cerebral blood flow (i.e. cerebral CT-perfusion) in association with additional neuromonitoring are necessary to understand the pathophysiology of neurological complications in ECMO patients and potentially help for their prevention.

DL cannulas in adults are large, because both the inflow and outflow cannulas must be accommodated within a single catheter. This provides the advantage in that only one vein needs to be accessed to provide full ECMO support to the patient. However, it is unclear whether large cannulas can be safely accommodated in the internal jugular veins without affecting cerebral blood flow dynamics. Cases of cerebral edema have been previously reported with internal jugular vein thrombosis, particularly when the contralateral internal jugular vein is hypoplastic or compromised [27]. In one small study of neurosurgical patients who had continuous intracranial pressure monitoring, cannulation of the right internal jugular vein was not associated with increases in intracranial pressure [28]. However, a previous case report indicated that bilateral cannulation in the internal jugular vein should be avoided, since it can increase the risk of intracranial hypertension due to impaired venous drainage. This could ultimately have repercussions on intracranial blood volume and pressure [29]. Recently, Sutter et al. [30] published a systematic review on neurological complication during VA and VV ECMO support, and they observed a similar proportion of neurological complications than in our study, with a higher incidence in patients treated with VA ECMO respect VV ECMO. Moreover, they observed a eightfold increased risk for AIS if the pre-ECMO lactates were above 10 mmol/L and a 18-fold increased risk for ICH in patients with thrombocytopenia. These additional factors should be taken into consideration for future studies dealing with the association of neurological complications and type of cannulation in ECMO patients.

Importantly, the findings of this study should be approached with caution, since there are several important limitations in our analysis. First, a number of centers use large return cannulas with DL cannulation (i.e. 25–27 French or greater) [31], so that the risk in terms of jugular vein obstruction would be similar in the two study groups. Unfortunately, the ELSO database does not contain data on cannula size for all patients, so we do not know how many patients in the SL group had large size return cannulas and could not adjust our analyses accordingly. Secondly, we do not know how many patients in each group had bilateral internal jugular vein cannulation; it is possible that a significant number of patients in both groups had a contralateral central venous catheter placed for vasoactive medication infusion or for other purposes and this might also contribute to alter the cerebral venous return. Thirdly, neurologic events are likely to be under-reported in the ELSO database because neurologic events during ECMO are often unrecognized or not confirmed. Obtaining magnetic resonance imaging scans is not feasible in ECMO patients and many patients are not taken for computed tomography scans because of the technical difficulties in moving ECMO patients or their inherent hemodynamic or respiratory instability. Forth, some other factors, including changes in PaCO_2_ after ECMO implementation, pre-ECMO bilirubin levels or the use of renal replacement therapy [32], have been associated with a higher risk for neurological complications in VV ECMO patients but were not available in our database. Finally, although reporting data to the ELSO database, it remains unknown whether participating centers had similar practices in managing ECMO patients as treatment variability might represent a significant confounder for our findings.

Our study also has important strengths. First, it is the largest study to examine whether DL ECMO cannulation is associated with an increased rate of adverse neurologic events. Furthermore, we used data from a well-established registry that has quality controls in place and represents a worldwide population. Finally, we used a propensity matching score to reduce imbalances between groups.

## Conclusions

Our findings showed that DL cannulation during V-V ECMO was not associated with an increased risk of neurological complications when compared with SL. Despite a higher survival rate in patients treated with DL, no differences in survival between the two cannula configurations were observed when patients with neurological injury were analyzed. Additional prospective studies should be encouraged to compare the effects of VV-ECMO cannulation on neurological events.

## Supplementary Information

**Additional file 1. Fig. S1**: A = Optimize plot; B = Overlap Assessment. **Fig. S2C** = Standardized effect size plot; D = Quantile–quantile (Q–Q) plot. **Fig. S3**: Model stratification by surgical technique, 8000 iterations.

## Data Availability

The datasets used and/or analyzed during the current study are available from the first author on reasonable request.

## Abbreviations

AIS: Acute ischemic stroke
CT: Computed tomography
DL: Double lumen
ECMO: Extra-corporeal membrane oxygenation
EEG: Electroencephalography
ELSO: Extracorporeal Life Support Organization
ICH: Intracranial hemorrhage
SL: Single lumen

## References

[1] Fan E, Gattinoni L, Combes A (2016). Venovenous extracorporeal membrane oxygenation for acute respiratory failure. Intensive Care Med.

[2] https://www.elso.org/registry/statistics/InternationalSummary.aspx

[3] Holzgraefe B, Broomé M, Kalzén H (2010). Extracorporeal membrane oxygenation for pandemic H1N1 2009 respiratory failure. Minerva Anestesiol.

[4] Lindén V, Palmér K, Reinhard J (2000). High survival in adult patients with acute respiratory distress syndrome treated by extracorporeal membrane oxygenation, minimal sedation, and pressure supported ventilation. Intensive Care Med.

[5] Luyt C-E, Bréchot N, Demondion P (2016). Brain injury during venovenous extracorporeal membrane oxygenation. Intensive Care Med.

[6] Arachchillage DRJ, Passariello M, Laffan M (2018). Intracranial hemorrhage and early mortality in patients receiving extracorporeal membrane oxygenation for severe respiratory failure. Semin Thromb Hemost.

[7] Mateen FJ, Muralidharan R, Shinohara RT (2011). Neurological injury in adults treated with extracorporeal membrane oxygenation. Arch Neurol.

[8] Lorusso R, Gelsomino S, Parise O (2017). Neurologic injury in adults supported with veno-venous extracorporeal membrane oxygenation for respiratory failure: findings from the extracorporeal life support organization database. Crit Care Med.

[9] Marinoni M, Migliaccio ML, Trapani S (2016). Cerebral microemboli detected by transcranial doppler in patients treated with extracorporeal membrane oxygenation. Acta Anaesthesiol Scand.

[10] Tulman DB, Stawicki SPA, Whitson BA (2014). Veno-venous ECMO: a synopsis of nine key potential challenges, considerations, and controversies. BMC Anesthesiol.

[11] Mazzeffi M, Kon Z, Menaker J (2018). Large dual-lumen extracorporeal membrane oxygenation cannulas are associated with more intracranial hemorrhage. ASAIO J Am Soc Artif Intern Organ.

[12] Liem KD, Kollée LA, Klaessens JH (1996). The influence of extracorporeal membrane oxygenation on cerebral oxygenation and hemodynamics in normoxemic and hypoxemic piglets. Pediatr Res.

[13] Menaker J, Tabatabai A, Rector R (2017). Incidence of cannula-associated deep vein thrombosis after veno-venous extracorporeal membrane oxygenation. ASAIO J Am Soc Artif Intern Organ.

[14] Camboni D, Philipp A, Lubnow M (2012). Extracorporeal membrane oxygenation by single-vessel access in adults: advantages and limitations. ASAIO J Am Soc Artif Intern Organs.

[15] Kuhl T, Michels G, Pfister R (2015). Comparison of the avalon dual-lumen cannula with conventional cannulation technique for venovenous extracorporeal membrane oxygenation. Thorac Cardiovasc Surg.

[16] Rubino A, Vuylsteke A, Jenkins DP (2014). Direct complications of the Avalon bicaval dual-lumen cannula in respiratory extracorporeal membrane oxygenation (ECMO): single-center experience. Int J Artif Organs.

[17] Hermon MM, Golej J, Mostafa G (2012). Veno-venous two-site cannulation versus veno-venous double lumen ECMO: complications and survival in infants with respiratory failure. Signa Vitae J Intesive Care Emerg Med.

[18] Ho DE, Imai K, King G, Stuart EA (2011). MatchIt: nonparametric preprocessing for parametric causal inference. J Stat Softw.

[19] Peluso L, Rechichi S, Franchi F, Pozzebon S, Scolletta S, Brasseur A, Legros B, Vincent JL, Creteur J, Gaspard N, Taccone FS (2020). Electroencephalographic features in patients undergoing extracorporeal membrane oxygenation. Crit Care.

[20] Abend NS, Dlugos DJ, Hahn CD, Hirsch LJ, Herman ST (2010). Use of EEG monitoring and management of non-convulsive seizures in critically ill patients: a survey of neurologists. Neurocrit Care.

[21] Hosokawa K, Gaspard N, Su F, Oddo M, Vincent JL, Taccone FS (2014). Clinical neurophysiological assessment of sepsis-associated brain dysfunction: a systematic review. Crit Care.

[22] Beumier M, Casu GS, Hites M, Wolff F, Cotton F, Vincent JL, Jacobs F, Taccone FS (2015). Elevated β-lactam concentrations associated with neurological deterioration in ICU septic patients. Minerva Anestesiol.

[23] Katyal N, Singh I, Narula N, Idiculla PS, Premkumar K, Beary JM, Nattanmai P, Newey CR (2020). Continuous electroencephalography (CEEG) in neurological critical care units (NCCU): a review. Clin Neurol Neurosurg.

[24] Javidfar J, Brodie D, Wang D (2011). Use of bicaval dual-lumen catheter for adult venovenous extracorporeal membrane oxygenation. Ann Thorac Surg.

[25] Skarsgard ED, Salt DR, Lee SK (2004). Extracorporeal life support organization registry: venovenous extracorporeal membrane oxygenation in neonatal respiratory failure: does routine, cephalad jugular drainage improve outcome?. J Pediatr Surg.

[26] O’Brien NF, Hall MW (2013). Extracorporeal membrane oxygenation and cerebral blood flow velocity in children. Pediatr Crit Care Med J Soc Crit Care Med World Fed Pediatr Intensive Crit Care Soc.

[27] Schummer W, Schummer C, Niesen W-D (2002). Unrecognized internal jugular vein obstruction: cause of fatal intracranial hypertension after tracheostomy?. J Neurosurg Anesthesiol.

[28] Woda RP, Miner ME, McCandless C, McSweeney TD (1996). The effect of right internal jugular vein cannulation on intracranial pressure. J Neurosurg Anesthesiol.

[29] Stocchetti N, Longhi L, Valeriani V (2003). Bilateral cannulation of internal jugular veins may worsen intracranial hypertension. Anesthesiol J Am Soc Anesthesiol.

[30] Sutter R, Tisljar K, Marsch S (2018). Acute neurologic complications during extracorporeal membrane oxygenation: a systematic review. Crit Care Med.

[31] Conrad SA, Wang D: Evaluation of recirculation during venovenous extracorporeal membrane oxygenation using computational fluid dynamics incorporating fluid–structure interaction. ASAIO J 2020; Epub ahead of print. 10.1097/MAT.0000000000001314 PMC8318564 33315664

[32] Cavayas YA, Munshi L, Del Sorbo L, Fan E (2020). The early change in PaCO_2_ after extracorporeal membrane oxygenation initiation is associated with neurological complications. Am J Respir Crit Care Med.

