# Supplementary material for: Neurological complications during veno-venous extracorporeal membrane oxygenation: Does the configuration matter? A retrospective analysis of the ELSO database
Source: Crit Care. 2021 Mar 17;25:107. doi: 10.1186/s13054-021-03533-5 (PMC7968168; doi:10.1186/s13054-021-03533-5)

**Supplemental Material**

1. ***How to Interpret the Balance Graphs***

**Supplemental Figure 1. Panel A = Optimize plot.** The plot is a graphical display of the balance criteria as a function of the GBM iteration. The graph demonstrates that a favorable balance was achieved with 1000 iterations and that a higher number of iterations that, thus were unnecessary. **Panel B = Overlap Assessment**. The figure presents the two sets of box plots of the distributions of propensity scores. An overlap between the groups is desirable meaning that there are no values of the pretreatment variables that occur only in one of the treatment conditions. However, there are no specific rules for what constitutes sufficient overlap so, in doubtful cases the combination of the overlap plot and the balance table are used. In the selected model, there is clearly a good overlap between the two boxplots.


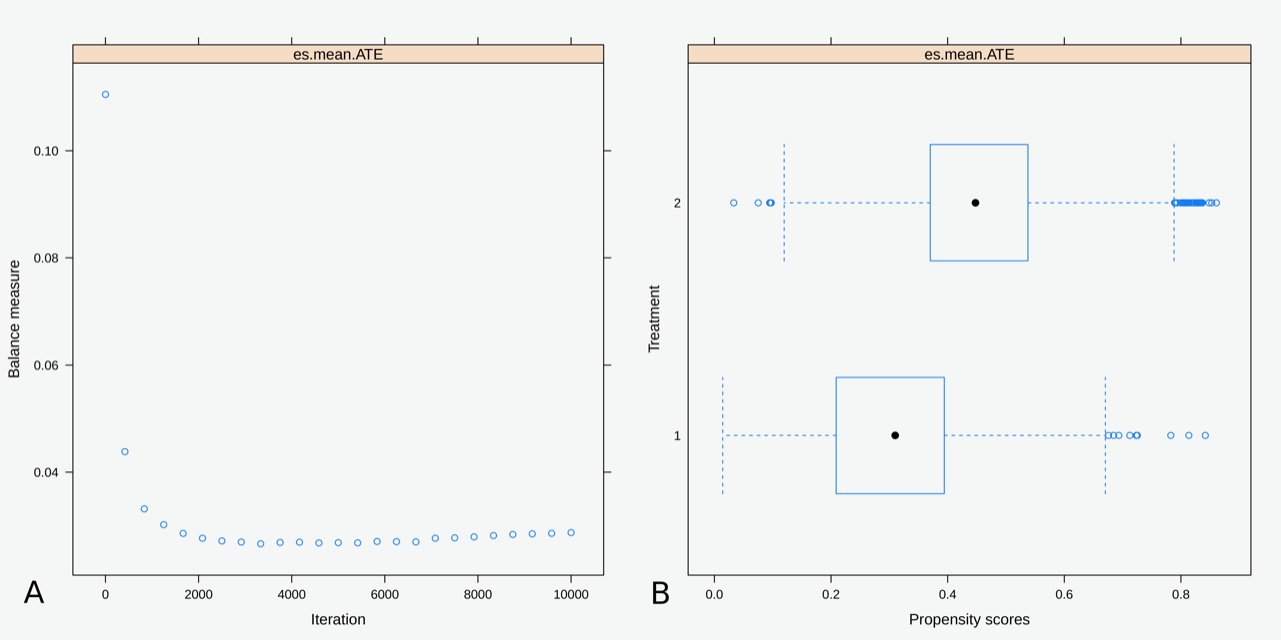


**Supplemental Figure 2: Panel C = Standardized Effect Size plot**. It assesses the balance of pretreatment variables before and after weighting. It shows the maximum pairwise absolute standardized mean differences (ASMDs). The ASMDs cutoff for defining unbalanced variables was 0.10. The light blue line represents pretreatment covariates for which the maximum pairwise ASMD reduced after weighting. The red lines mean the pretreatment covariates for which the maximum pairwise ASMD increased after weighting. A good balance is obtained when, after weighting in the majority of variables the ASMDs are lower than 0.10 and there is a prevalence of light blue lines. The weighted ASMD values were lower than 0.10 in all cases. **Panel D =** **Quantile‐quantile (Q‐Q) plot.** This plot also assesses the balance of pretreatment variables. In the plot, the Kolmogorov-Smirnov p-value is plotted against the rank of p-value for pretreatment variables. Along a 45-degree fitting line, open symbols represent weighted covariates and solid symbols represent unweighted covariates. A good balance is obtained when open symbols lie close, below or above, the 45-degree line. Before weighting, all the values were below the 45‐degree line. A good balance was achieved after weighting, with p-values that lie around the 45‐degree line.


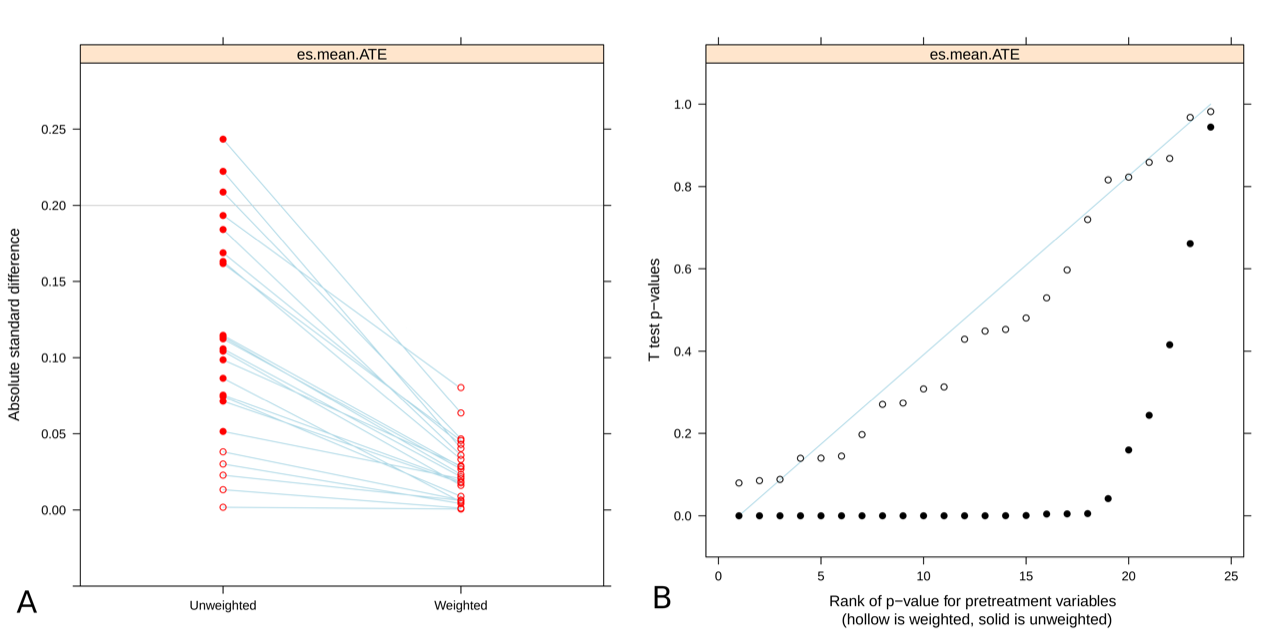


**Supplemental Figure 3: Model stratification by surgical technique, 8000 iterations.** **Panel A = Standardized Effect Size plot.** The weighted ASMD values were lower than 0.20 in all cases. **Panel B = Quantile‐quantile (Q‐Q).** Before weighting, most of the values were below the 45‐degree line. A good balance was achieved after weighting, with p-values that lie close the 45‐degree line.


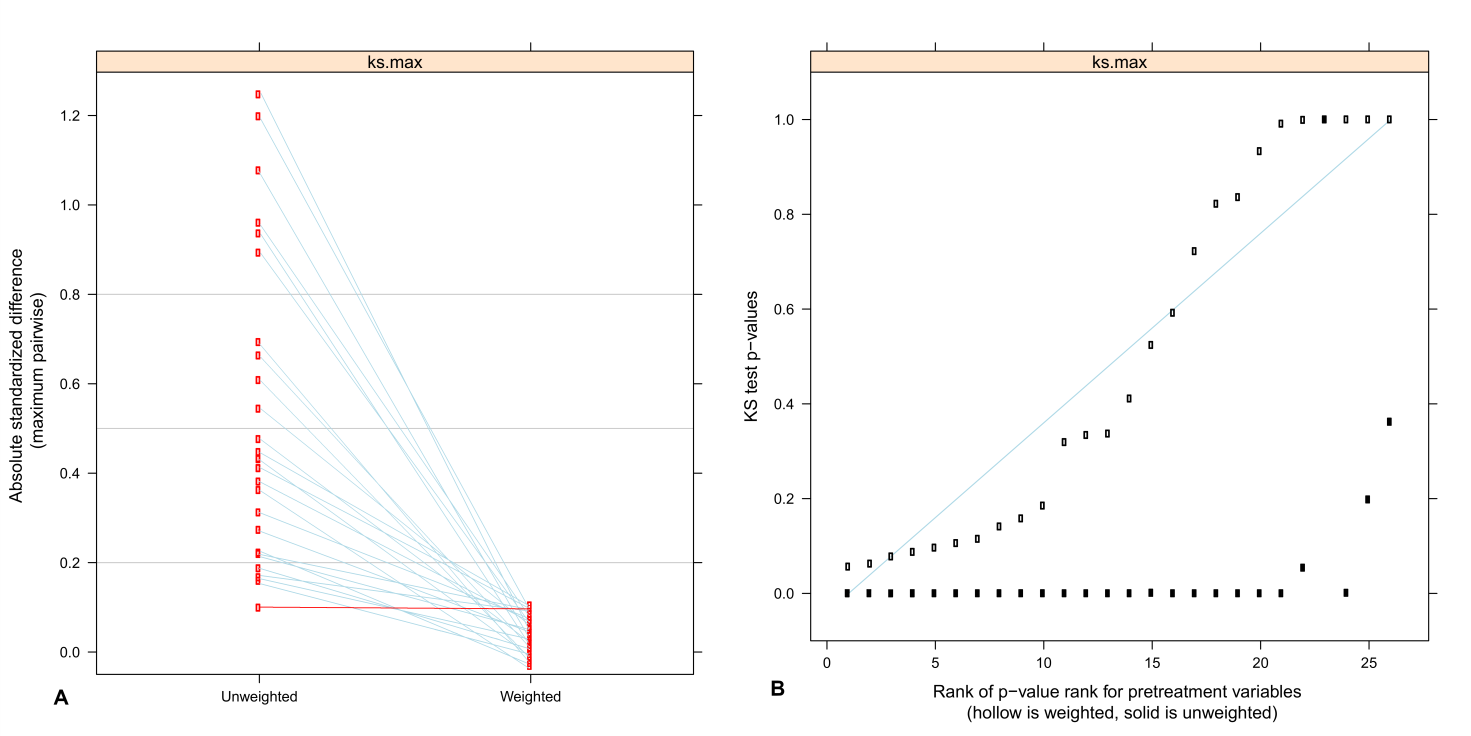

Supplement: Supplementary file 1 — Additional file 1. Fig. S1: A = Optimize plot; B = Overlap Assessment. Fig. S2C = Standardized effect size plot; D = Quantile–quantile (Q–Q) plot. Fig. S3: Model stratification by surgical technique, 8000 iterations. [file 13054_2021_3533_MOESM1_ESM.docx]
